# Comparison of transoral robotic surgery with other surgeries for obstructive sleep apnea

**DOI:** 10.1038/s41598-020-75215-1

**Published:** 2020-10-23

**Authors:** Yung-An Tsou, Wen-Dien Chang

**Affiliations:** 1 Department of Otolaryngology-Head and Neck Surgery, China Medical University Hospital, Taichung, Taiwan; 2 Department of Audiology and Speech Pathology, Asia University, Taichung, Taiwan; 3 Department of Sport Performance, National Taiwan University of Sport, No.16, Sec. 1, Shuang-Shih Rd, Taichung, 40404 Taiwan

**Keywords:** Surgery, Therapeutics

## Abstract

This study compared the characteristic surgical parameters and clinical effects of transoral robotic surgery (TORS) and other available methods used to alleviate obstructive sleep apnea. Articles on TORS and other surgeries for obstructive sleep apnea were identified in the PubMed and EMBASE databases. Two investigators independently reviewed the articles and classified the data for meta-analysis. The pooled effect sizes of TORS (standardized mean difference; SMD = − 2.38), coblation tongue base resection (CTBR; SMD = − 2.00) and upper airway stimulation (UAS; SMD = − 0.94) revealed significant improvement in the apnea–hypopnea index (AHI; p < 0.05). The lowest O_2_ saturation reported was significantly increased following TORS (SMD = 1.43), CTBR (SMD = 0.86) and UAS (SMD = 1.24, p < 0.05). Furthermore, TORS (SMD = − 2.91) and CTBR (SMD = − 1.51, p < 0.05) significantly reduced the Epworth Sleepiness Scale (ESS) score. No significant difference in operation time, success rate, or instances of complication were observed between TORS and the other compared interventions. The use of TORS in obstructive sleep apnea has the same rate of success and failure as other methods of surgical intervention for obstructive sleep apnea with no statistical difference in operation times. The reported clinical effects on the AHI, lowest O_2_ saturation, and ESS scores of TORS were similar to those of other surgeries.

## Introduction

The number of reported cases of obstructive sleep apnea is increasing. A key factor causing sleep apnea is the narrowing of oropharyngeal space, termed retroglossal and retropalatal airway narrowing^1^. Therefore, surgical methods that target the base of the tongue are especially useful in managing a narrowed oropharynx. The available methods for treating a bulky tongue base include radiofrequency, coblation^2^, transoral robotic surgery^3^, transoral robotic laser surgery^4^, tongue suspension, hyoid suspension surgery^5^, and hypoglossal nerve stimulation^3^. Clinicians and sleep surgeons must employ several diagnostic tools when deciding whether to treat the base of the tongue with surgery^6^. These tools include physical examinations, Muller tests using awake laryngoscopy^7^, drug-induced sleep endoscopy^8^, sleep computed tomography or sleep magnetic resonance imaging, cephalometry and series X-ray assessments^9^. The information obtained from a combination of examinations enables the development of a unique surgical strategy for each patient^10^. Although no single treatment method is currently effective for every patient, several operations have obtained comparable outcomes in treating sleep apnea. This study aimed to perform a systematic review and meta-analysis of the clinical effects and characteristic surgical parameters of transoral robotic surgery (TORS) compared with other surgical interventions for obstructive sleep apnea. The results of this analysis can serve to inform surgeons managing sleep apnea related to the base of the tongue.

## Material and methods

### Articles search

**Figure 1:**
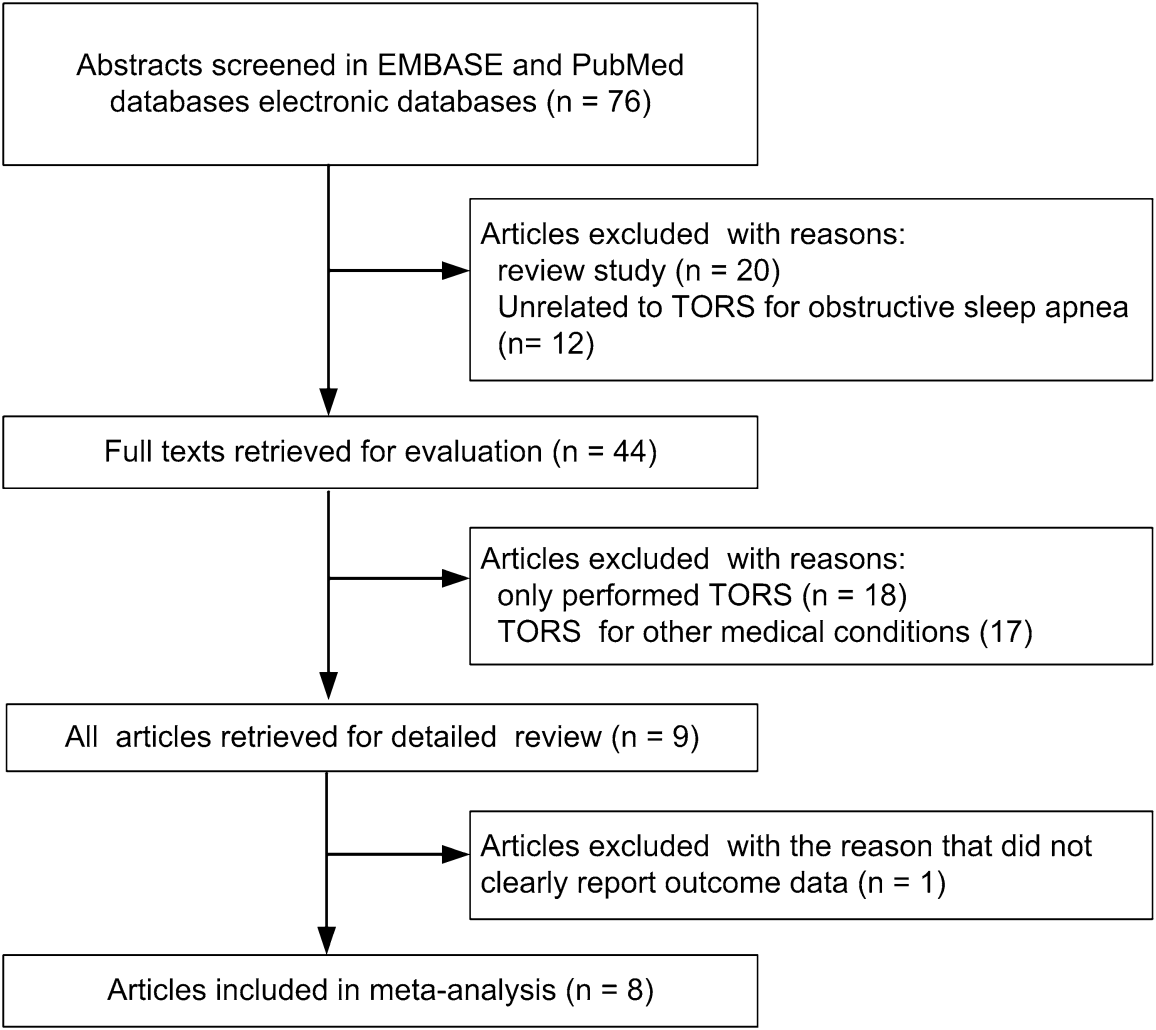
Flowchart of study selection.

The PubMed and EMBASE databases were used searched for articles. The following search strategies were used for the PubMed database: #1 ("obstructive sleep apnea"[All Fields] OR "transoral robotic surgery"[All Fields]), #2 ("robotic surgical procedures "[MeSH Terms] OR "sleep apnea syndromes"[MeSH Terms]), #3 ("surgery" OR "sleep apnea, obstructive"[MeSH Terms]), and #4 (#1, #2AND #3). The following search strategies were used for the EMBASE database: #1 (“obstructive sleep apnea”'/exp OR “transoral robotic surgery”), #2 (“sleep apnea syndromes” OR “surgery”), and #3 (#1 AND #2). The references within the searched-for articles to provide additional data. The article search strategy was based on the Preferred Reporting Items for Systematic Reviews and Meta-Analyses (PRISMA) statement (Fig. 1).

### Study selection criteria

The articles that compared TORS with various methods were included in the present study and their data enrolled for meta-analysis. Each procedure was evaluated on the basis of the following criteria: apnea–hypopnea index (AHI), lowest O_2_ saturation, Epworth Sleepiness Scale (ESS) score, complication rate, and success rate. The articles included in this study were human studies written in English that detailed pre-surgery and post-surgery outcomes of TORS compared with other surgical interventions. Articles without data on surgical outcomes, studies on only one particular operation, and reviews on TORS as a treatment for unrelated medical conditions were all excluded. Severity of sleep apnea was diagnosed using attended overnight polysomnography for each patient in the eight studies included, and the scoring system was defined by the American Academy of Sleep Medicine (AASM)^4,11–17^. Babademez et al. used the AASM 2.2 edition^12^, and Hwang et al. and Lan et al. used the AASM 2.5 edition to measure overnight PSG^11,13^. The other referenced studies scored patients using the AASM 2nd edition^4,14–17^. Pre-operative and post-operative apnea were defined as more than 10 s of no or decreased oronasal airflow. Hypopnea was defined as a discernible fall in oronasal flow with SpO_2_ desaturation. All the included studies used the AASM scoring manual for evaluation. Therefore, the obstructive sleep apnea severity and surgical outcomes are comparable.

### Data extraction and analysis

Two investigators independently reviewed the articles and obtained study design, intervention method comparison, study size, and clinical outcomes data. Surgical outcome data were divided into the following categories: AHI, lowest O_2_ saturation, and ESS score. Furthermore, data regarding surgery efficiency and efficacy were collected, including operation time, complication rate, and success rate. The quality of data obtained from the articles was determined through the Quality Assessment of Diagnostic Accuracy Studies-2 (QUADAS-2) tool. A funnel plot was used to assess publication bias. The success rate, defined as AHI < 20 events/h after treatment and ≥ 50% reduction of pre-operative AHI, and heterogeneity were studied through meta-analysis.

### Statistical analysis

All data were analyzed using MedCalc 14 (MedCalc Software, Oostende, Belgium). The continuous variables (AHI, lowest O_2_ saturation, and ESS score) were collected for both the pre-intervention and post-intervention states. Standardized mean differences (SMD) with 95% confidence intervals (CI) were used to analyze the effect sizes for each study. Pooled SMD with 95% CIs were estimated across the same interventions. TORS was compared with alternative interventions for obstructive sleep apnea, and SMDs with 95% CIs were used to compare continuous variables of surgery efficiency. Moreover, the success and complication rates, which were categorical variables, were assessed using the odds ratio and relative risk, respectively. The I^2^ statistic for heterogeneity was employed to estimate fixed or random effect models. A value of p < 0.05 was considered statistically significant.

## Results

**Figure 2:**
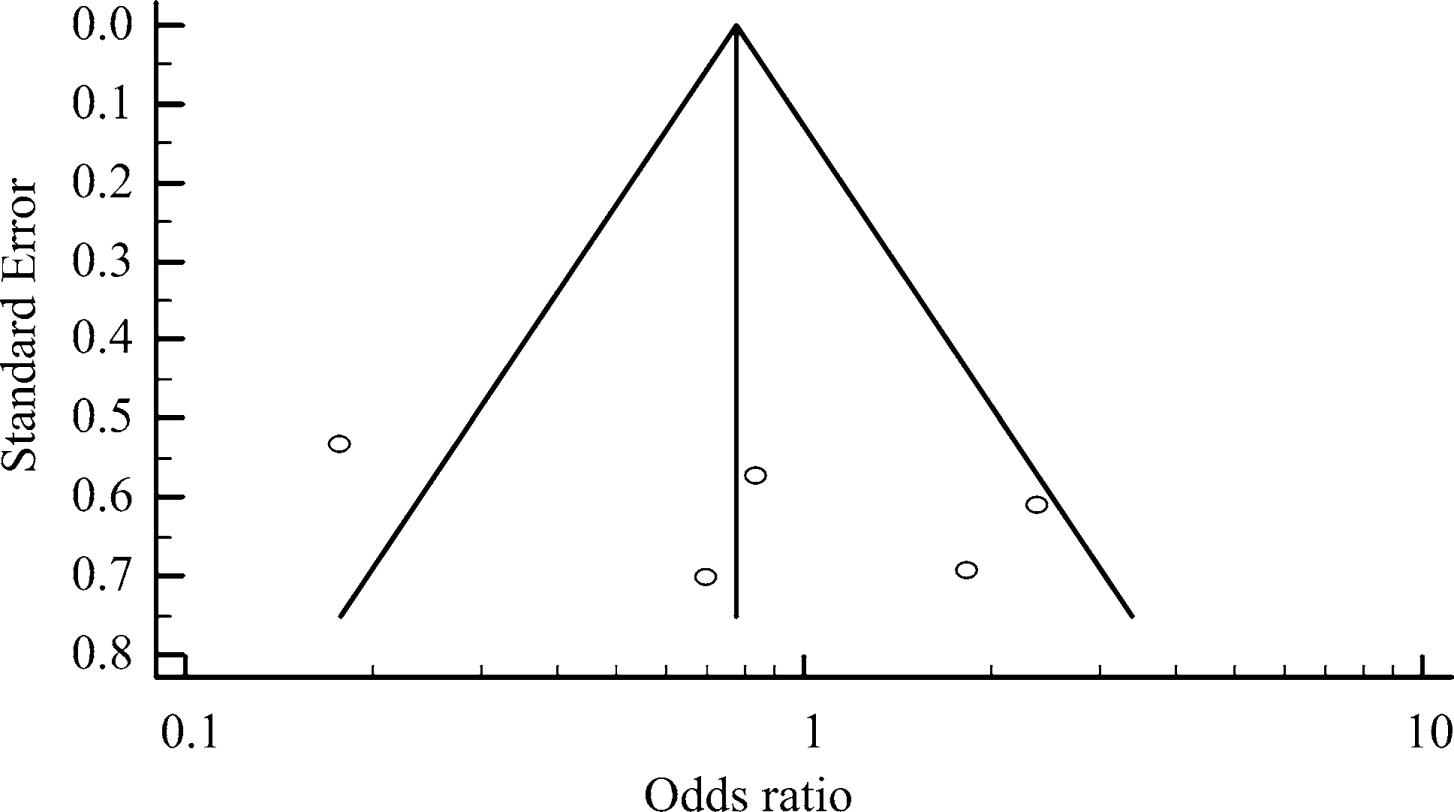
Funnel plot analysis of publication bias in included studies.

In January 2020, 76 articles were identified in a preliminary search, and 8 of these articles met the criteria for review^4,11–17^. The results of QUADAS-2 assessment of the included articles are illustrated in Table 1. A funnel plot was used to examine bias for meta-analysis (Fig. 2) and revealed a significant treatment effect in these studies (p < 0.05). Among the included studies, six were retrospective^11–15,17^, one had a pre-post-test design^16^, and one was prospective^4^. In total, 188 patients with obstructive sleep apnea treated with TORS and 230 cases treated with alternative interventions were included in the analysis. Three studies investigated coblation tongue base resection (CTBR, total n = 79)^11–13^, and two studies investigated upper airway stimulation (UAS, total n = 105)^14,15^. Three articles reported on radiofrequency (n = 20), CO_2_ laser (n = 10), and endoscopic partial midline glossectomy (EPMG, n = 16)^4,16,17^. The success numbers of TORS and alternative interventions are represented in Table 2. However, the post-operation cure rate in the article of Yu et al. was defined as AHI < 5^15^, whereas other studies used the success rate.

**Table 1:** The results of QUADAS-2 in included studies.

Author (year)	Risk of bias				Applicability concerns		
	Patient selection	Index test	Reference standard	Flow and timing	Patient selection	Index test	Reference standard
Hwang (2019)^11^				?			
Babademez (2019)^12^				?			
Lan (2019)^13^				?			
Huntley (2019)^14^				?			
Yu (2019)^15^				?			
Aynacı (2018)^16^		?					
Karaman (2017)^4^				?			
Folk (2017)^17^				?			

, low risk;  , high risk, ?, unclear risk.

**Table 2:** Overview of studies on TORS and compared studies.

Author (year)	Design	Intervention (n)	Obstructive sleep apnea patients			Success after intervention (n)	Clinical outcomes
			Age	Gender (M/F)	BMII (kg/m^2^)		
Hwang (2019)^11^	Retrospective	TORS (16)	45.1 ± 13.4	14/2	25.8 ± 3.4	12	Decrease AHI* and ESS, and increase lowest O_2_ saturation*
		CTBR (29)	39.8 ± 10.8	26/3	26.8 ± 2.8	18	Decrease AHI* and ESS, and increase lowest O_2_ saturation*
Babademez (2019)^12^	Retrospective	TORS (37)	40.9 ± 9.2	28/9	27.0 ± 4.4	28	Decrease AHI* and ESS*
		CTBR (33)	39.4 ± 8.5	25/8	28.3 ± 3.9	26	Decrease AHI* and ESS*
Lan (2019)^13^	Retrospective	TORS (16)	39.4 ± 12.3	15/1	28.2 ± 3.8	8	Decrease AHI* and ESS*, and increase lowest O_2_ saturation*
		CTBR (17)	38.7 ± 11.5	13/4	27.4 ± 5.6	10	Decrease AHI* and ESS*, and increase lowest O_2_ saturation*
Huntley (2019)^14^	Retrospective	TORS (24)	46.4 ± 13.9	20/4	29.6 ± 3.8	13	Decrease AHI and ESS, and increase lowest O_2_ saturation
		UAS(76)	61.9 ± 12.0	50/26	29.4 ± 4.1	66	Decrease AHI and ESS, and increase lowest O_2_ saturation
Yu (2019)^15^	Retrospective	TORS(20)	53^a^	16/4	< 35	15^b^	Decrease AHI and ESS, and increase lowest O_2_ saturation
		UAS(29)	62^a^	25/4	< 35	23^b^	Decrease AHI and ESS, and increase lowest O_2_ saturation
Aynacı (2018)^16^	Pre-post test	TORS(20)	45.0 ± 7.1	16/4	NA	20	Decrease AHI* and ESS*, and increase lowest O_2_ saturation*
		Radiofrequency(20)	41.7 ± 8.4	17/3	NA	20	Decrease AHI* and ESS*, and increase lowest O_2_ saturation*
Karaman (2017)^4^	Prospective	TORS(10)	45.1 ± 7.2	16/4	NA	10	Decrease AHI and ESS, and increase lowest O_2_ saturation
		CO_2_ laser(10)				10	Decrease AHI and ESS, and increase lowest O_2_ saturation
Folk (2017)^17^	Retrospective	TORS(45)	48.2 ± 11.6	33/12	32.3 ± 4.8	34	Decrease AHI* and ESS*, and increase lowest O_2_ saturation*
		EPMG(16)	46.3 ± 8.4	12/4	32.5 ± 3.5	9	Decrease AHI and ESS, and increase lowest O_2_ saturation

*NA*, not available; *M/F*, male/female; *BMI*, body mass index; *TORS*, transoral robotic surgery; *CTBS*, coblation tongue base resection; *UAS*, upper airway stimulation; *EPMG*, endoscopic partial midline glossectomy; *AHI*, apnea–hypopnea index; *ESS*, Epworth Sleepiness Scale.

^a^Average age; ^b^ cure no. of patients; *Statistical significance in pre- and post-operation (p < 0.05).

### AHI, lowest O_2_ saturation, and ESS score after TORS and alternative interventions

Preoperation and postoperation AHIs were reported in all the papers (Table 2)^4,11–17^. All patients treated with TORS displayed a significant decrease in AHI (p < 0.05), and the total random effect was − 2.38 (a decline in average AHI from 41.56 to 17.10 events/h), as displayed in Table 3. The pooled effect sizes of CTBR (a decline in average AHI from 42.92 to 17.42 events/h; total random effect = − 2.00) and UAS (a decline in average AHI from 38.58 to 7.21 vents/h; total fixed effect = − 0.94) also revealed significant improvement in AHI (p < 0.05). Aynacı et al., Karaman et al., and Folk et al. reported mean effects sizes on the AHI of − 2.42, − 5.96 and − 0.66, respectively^4,16,17^. These studies did not include pooled effect sizes of AHI for radiofrequency, CO_2_ laser or EPMG.

**Table 3:** Effect sizes on AHI among the interventions.

Author (year)	Intervention	n	Weight (%)	SMD	SE	95% CI	p
Hwang (2019)^11^	TORS	16	11.42	− 1.82	0.41	− 2.66 to − 0.97	
Babademez (2019)^a,^^12^	TORS	37	12.01	− 2.71	0.32	− 3.34 to − 2.07	
Babademez (2019)^b,^^12^	TORS	37	12.06	− 2.55	0.31	− 3.17 to − 1.93	
Lan (2019)^13^	TORS	16	11.65	− 1.24	0.37	− 2.01 to 0.47	
Huntley (2019)^14^	TORS	24	12.17	− 0.66	0.29	− 1.25 to − 0.08	
Yu (2019)^15^	TORS	20	11.89	− 1.26	0.34	− 1.95 to − 0.57	
Aynacı (2018)^16^	TORS	20	8.16	− 7.06	0.84	− 8.78 to − 5.34	
Karaman (2017)^4^	TORS	10	8.21	− 4.58	0.84	− 6.34 to − 2.81	
Folk (2017)^17^	TORS	45	12.43	− 1.63	0.24	− 2.11 to − 1.15	
Total random effect	TORS	225	100.00	− 2.38	0.40	− 3.15 to − 1.59	0.001
**Heterogeneity test**			Q = 83.29, df = 8, I^2^ = 90.40%				0.001
Hwang (2019)^11^	CTBR	29	25.56	− 1.64	0.30	− 2.24 to − 1.04	
Babademez (2019)^a,^^12^	CTBR	33	24.49	− 3.12	0.36	− 3.85 to − 2.40	
Babademez (2019)^b,^^12^	CTBR	33	25.12	− 2.52	0.32	− 3.18 to − 1.87	
Lan (2019)^13^	CTBR	17	24.82	− 0.72	0.34	− 1.42 to − 0.01	
Total random effect	CTBR	112	100	− 2.00	0.50	− 2.99 to − 1.01	0.001
**Heterogeneity test**			Q = 27.13, df = 3, I^2^ = 88.94%				0.001
Huntley (2019)^14^	UAS	76	68.82	− 0.80	0.23	− 1.27 to -0.33	
Yu (2019)^15^	UAS	29	31.18	− 1.25	0.35	− 1.97 to − 0.53	
Total fixed effect	UAS	105	100	− 0.94	0.19	− 1.33 to − 0.55	0.001
**Heterogeneity test**			Q = 1.10, df = 1, I^2^ = 9.48%				0.29
Aynacı (2018)^16^	Radiofrequency	20	NA	− 2.42	0.41	− 3.25 to − 1.58	
Karaman (2017)^4^	CO2 laser	10	NA	− 5.96	1.03	− 8.14 to − 3.79	
Folk (2017)^17^	EPMG	16	NA	− 0.66	0.35	− 1.39 to 0.05	

*SMD* standardized mean difference, *SE* standard error, *CI* confidence intervals, *TORS* transoral robotic surgery, *CTBS* coblation tongue base resection, *UAS* upper airway stimulation, *EPMG* endoscopic partial midline glossectomy.

Six studies measured the lowest O_2_ saturation before and after the operation^11,13–17^. As illustrated in Table 4, a comparison of SMD in TORS revealed a significant increase in lowest O_2_ saturation (increase in average lowest O_2_ saturation from 79.83 to 86.81%; total random effect = 1.43, p < 0.05). The pooled effect sizes for CTBR (increase in average lowest O_2_ saturation from 76.25 to 83.01%; total fixed effect = 0.86) and UAS (an increase in average lowest O_2_ saturation from 79.45 to 85.55%; total fixed effect = 1.24) also revealed significant increases in lowest O_2_ saturation (p < 0.05). Only one study on radiofrequency^16^ and one on EPMG^17^ reported that of lowest O_2_ saturation was improved, and the effect sizes were 2.57 and 0.32, respectively.

**Table 4:** Effect sizes on lowest O_2_ saturation among the interventions.

Author (year)	Intervention	n	Weight (%)	SMD	SE	95% CI	p
Hwang (2019)^11^	TORS	16	17.18	0.81	0.35	0.07 to 1.54	
Lan (2019)^13^	TORS	16	16.85	1.56	0.39	0.75 to 2.37	
Huntley (2019)^14^	TORS	24	17.76	0.36	0.28	− 0.21 to 0.93	
Yu (2019)^15^	TORS	20	17.21	0.23	0.35	− 0.50 to 0.96	
Aynacı (2018)^16^	TORS	20	12.75	6.52	0.79	4.92 to 8.13	
Folk (2017)^17^	TORS	45	18.25	0.51	0.21	0.09 to 0.93	
Total random effect	TORS	141	100	1.43	0.49	0.45 to 2.41	0.004
**Heterogeneity test**			Q = 62.50, df = 9, I2 = 92%				0.001
Hwang (2019)^11^	CTBR	29	60.36	1.06	0.27	0.50 to 1.61	
Lan (2019)^13^	CTBR	17	39.64	0.57	0.34	− 0.12 to 1.27	
Total fixed effect	CTBR	46	100	0.86	0.21	0.44 to 1.29	0.001
**Heterogeneity test**			Q = 1.22, df = 1, I2 = 18.32%				0.26
Huntley (2019)^14^	UAS	76	74.74	1.33	0.17	0.98 to 1.68	
Yu (2019)^15^	UAS	29	25.26	0.978	0.30	0.35 to 1.59	
Total fixed effect	UAS	105	100	1.24	0.15	0.93 to 1.54	0.001
**Heterogeneity test**			Q = 1.22, df = 1, I2 = 18.32%				0.31
Aynacı (2018)^16^	Radiofrequency	20	NA	2.57	0.42	1.71 to 3.43	
Folk (2017)^17^	EPMG	16	NA	0.32	0.34	− 0.38 to 1.02	

*SMD* standardized mean difference, *SE* standard error, *CI* confidence intervals, *TORS* transoral robotic surgery, *CTBS* coblation tongue base resection, *UAS* upper airway stimulation, *EPMG* endoscopic partial midline glossectomy.

As displayed in Table 5, five studies measured ESS score before and after the operation^11–13,16,17^. Comparison of the effect size after treatment with TORS revealed a significant decrease in ESS scores (decline in average ESS score from 12.82 to 5.33; total random effect = − 2.91, p < 0.05). Comparison of the pre-operative and post-operative ESS scores in three studies revealed that CTBR caused a significant decrease (decline in average ESS score from 10.66 to 6.43; total random effect = − 1.51, p < 0.05). However, the ESS scores of patients treated with CTBR were higher than those of patients treated with TORS. One article on radiofrequency^16^ and one article on EPMG^17^ revealed a reduction in ESS score at − 4.19 and − 0.83, respectively.

**Table 5:** Effect sizes on ESS among the interventions.

Author (year)	Intervention	n	Weight (%)	SMD	SE	95% CI	p
Hwang (2019)^11^	TORS	16	21.02	− 0.92	0.36	− 1.66 to − 0.19	
Babademez (2019)^12^	TORS	37	21.01	− 3.39	0.36	− 4.11 to − 2.67	
Lan (2019)^13^	TORS	16	21.02	− 0.81	0.35	− 1.54 to − 0.08	
Aynacı (2018)^16^	TORS	20	15.43	− 9.06	1.06	− 11.21 to − 6.92	
Folk (2017)^17^	TORS	45	21.52	− 2.01	0.25	− 2.52 to − 1.50	
Total random effect	TORS	134	100	− 2.91	0.76	− 4.40 to − 1.41	0.001
**Heterogeneity test**			Q = 78.97, df = 4, I^2^ = 94.93%				0.001
Hwang (2019)^11^	CTBR	29	33.92	− 0.51	0.26	− 1.04 to 0.01	
Babademez (2019)^12^	CTBR	33	32.79	− 3.57	0.39	− 4.35 to − 2.78	
Lan (2019)^13^	CTBR	17	33.30	− 0.51	0.34	− 1.20 to 0.18	
Total random effect	CTBR	79	100.00	− 1.51	0.92	− 3.33 to 0.30	0.10
**Heterogeneity test**			Q = 46.91, df = 2, I^2^ = 95.74%				0.001
Aynacı (2018)^16^	Radiofrequency	20	NA	− 4.19	0.56	− 5.33 to − 3.05	
Folk (2017)^17^	EPMG	16	NA	− 0.83	0.36	− 1.56 to − 0.09	

*SMD* standardized mean difference, *SE* standard error, *CI* confidence intervals, *TORS* transoral robotic surgery, *CTBR* coblation tongue base resection, *EPMG* endoscopic partial midline glossectomy.

### Operation time in TORS and compared interventions

**Figure 3:**
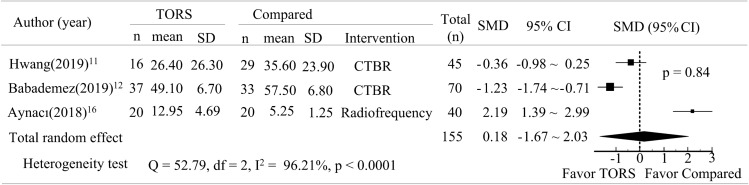
Forest plot depicting operation time between TORS and compared intervention.

Three articles reported data on operation time^11,12,16^. A comparison of effect size between TORS and the alternative interventions, CTBR and radiofrequency, revealed no significant differences in operation time for the treatment of obstructive sleep apnea (p > 0.05, Fig. 3).

### Success rate and complication rate in TORS and compared interventions

**Figure 4:**
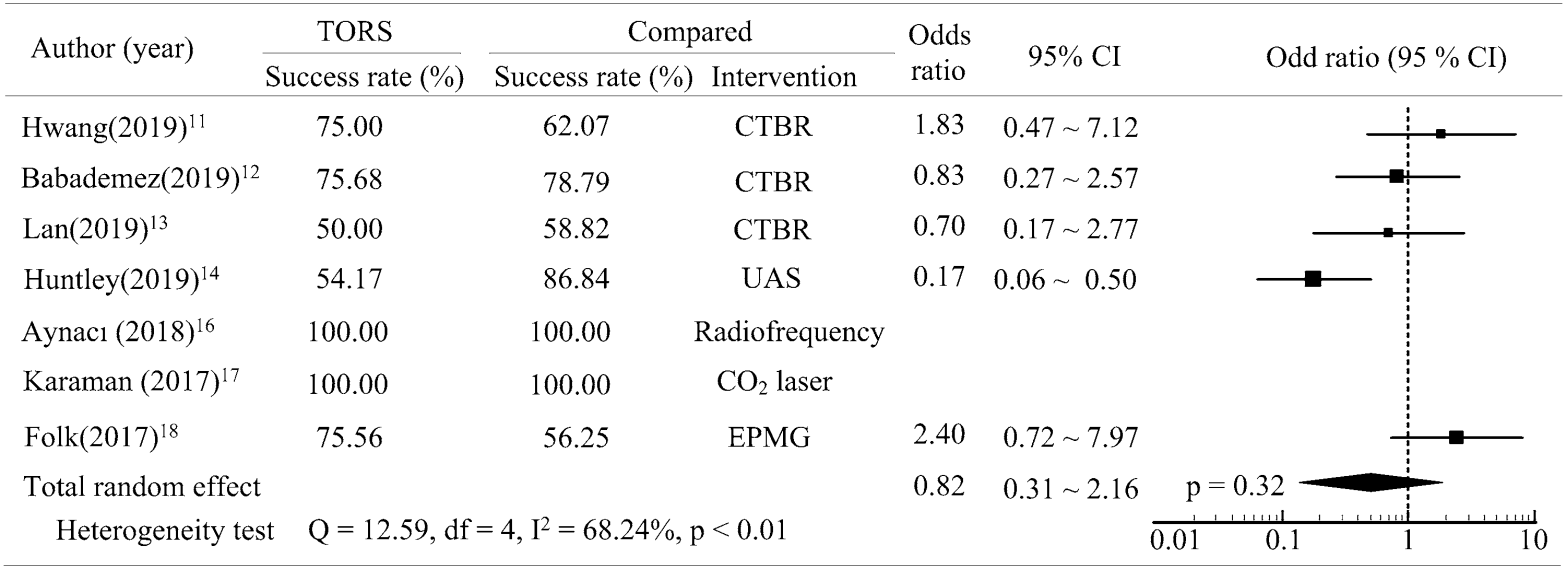
Forest plot depicting success rate between TORS and compared intervention.

**Figure 5:**
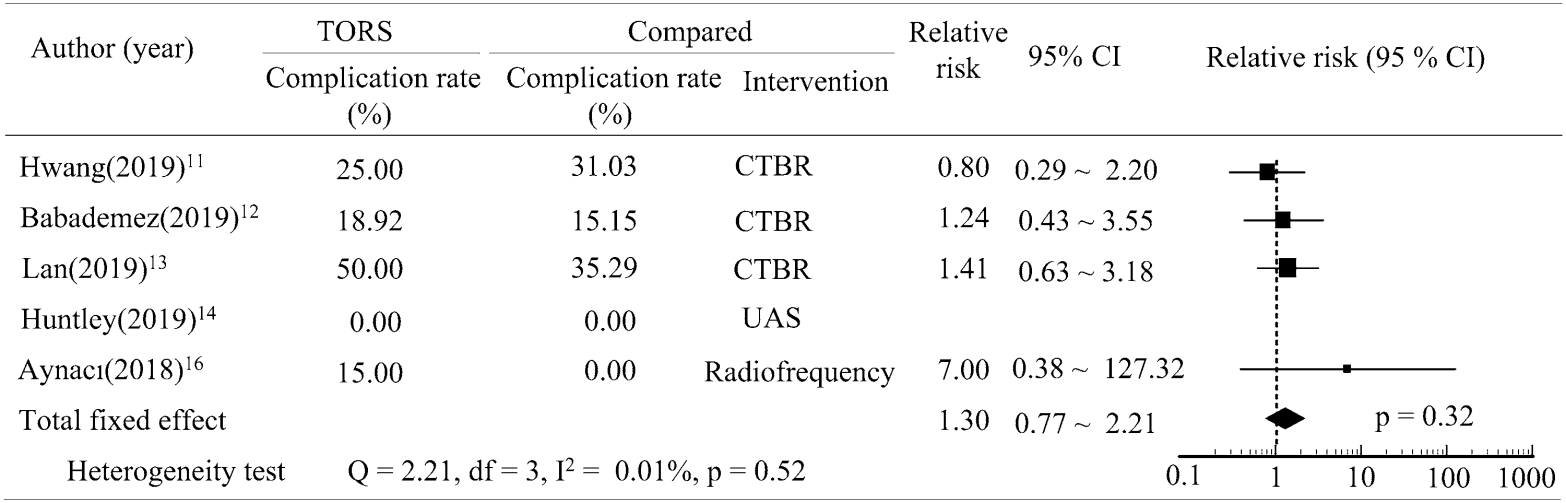
Forest plot depicting complication rate between TORS and compared intervention.

Seven studies compared the success rate of TORS with that of alternative interventions (CTBR, UAS, radiofrequency, CO_2_ laser and EPMG)^4,11–14,16,17^. The odds ratio revealed that no significant differences were observed in the success rates using TORS and other interventions for obstructive sleep apnea (pooled odds ratio = 0.82, p > 0.05, Fig. 4). Five studies reported the complication rates after treatment with TORS or a comparedintervention (CTBR, UAS, or radiofrequency)^11–14,16^. An assessment of the relative risk revealed that the complication rate of TORS was higher than those of the alternative interventions. However, this difference was also non-significant (pooled relative risk = 1.30, p > 0.05, Fig. 5).

## Discussion

TORS is a novel and technologically advanced surgery that has been used to treat obstructive sleep apnea. This systematic review was conducted to compare the clinical effects of the surgical interventions, such as TORS, CTBR, UAS, radiofrequency, CO_2_ laser, and EPMG. Clinical outcome (AHI, lowest O2 saturation, and ESS score) and surgical effect (operation time, success rate, and complication rate) data for TORS and alternative interventions were compared using meta-analysis. The eight published articles included in our analysis are summarized in Table 2. Our results demonstrated that TORS, and the other interventions, significantly reduced the AHI and ESS score and increased the lowest O_2_ saturation in patients with obstructive sleep apnea patients. No significant differences in operation time, success rate, or complication rate were observed between TORS and the compared interventions (p > 0.05).

A meta-analysis of the procedures revealed that the pooled SMD in the AHI, lowest O_2_ saturation, and ESS score for TORS were larger than those for CTBR and UAS. All three interventions had significant effects on obstructive sleep apnea (p < 0.05). However, UAS is currently considered to be the optimal method for widening the oropharynx and retroglossal space^18^. The Food and Drug Administration approves of UAS in patients with tongue base anterior–posterior collapse and an AHI of < 50/h^19^. Huntley et al. indicated that the UAS surgical procedure is a multilevel procedure and focuses on tongue base obstruction and airway caliber in patients with obstructive sleep apnea. CTBR has the advantages of minimal training time and lower cost than TORS^14^. However, the excitement of electrodes on the instrument can create ionized particles in saline solution and disrupt intercellular bonds. The outcomes of CTBR show unfavorable effects of necrosis and scar tissue formation in patients who underwent the procedure^20^. However, in our systematic review, we found that patients treated with TORS had reduction in both AHI and lowest O_2_ saturation values as well as ESS improvement, and the effect size of TORS was slightly superior to those of CTBR and UAS. We thought that this is because TORS is performed using a real layer resection of lingual tonsils and a partial resection of tongue base muscle. Therefore, the volume reduction is more prominent than the volume reduction following radiofrequency or CTBR. Radiofrequency and CTBR may exhibit a firewall effect, meaning that the volume-reduction effects may be minimized by denatured tissue blocking energy transfer from the core of the surgical devices. Furthermore, UAS electric titration is not sufficiently powerful for most cases of huge tongue collapse or lateral pharyngeal collapse. The rate of increased volume varies depending on the quantity of electric power used, which also explains the unstable improvements in AHI, lowest O_2_ saturation, and ESS score.

We identified few data comparisons of AHI, lowest O_2_ saturation and ESS score following treatment with radiofrequency, CO_2_ laser, and EPMG. The radiofrequency method reduces tissue volume using high-frequency current flow to apply a selective temperatures^21^. Patients with obstructive sleep apnea had decreased snoring and improved respiratory effort when treated with radiofrequency applied to the soft palate^22^. The results reported by Aynacı et al. indicated that the radiofrequency method had better outcomes than TORS in terms of operation duration, length of post-operation hospitalization, and oral feeding duration^16^. Furthermore, 15% of complications from all methods were found after treatment with TORS, while no complications occurred after treatment with radiofrequency. CO_2_ laser treatment is a cost-effective surgery that causes less thermal damage from tissue cutting and coagulation than alternative methods^23^. Karaman et al. determined that the CO_2_ laser method has a lower complication rate, operation duration, length of postoperative hospitalization, and oral feeding time than TORS. After treatment with CO_2_ laser, patients with sleep apnea patients had a significantly lower AHI and significantly less postoperative pain (p < 0.05)^4^. Furthermore, tissue resection is easier using CO_2_ laser treatment compared with TORS^4,24^. EPMG was reported to be an effective surgical procedure in treating obstructive sleep apnea^25^. Folk et al. compared the changes in AHI, lowest O_2_ saturation, and ESS score pre- and post-operation, but no significant differences were observed (p > 0.05)^17^. TORS has been demonstrated to have superior clinical effects to EPMG in patients with sleep apnea. Few studies have compared TORS to methods such as radiofrequency, CO_2_ laser, and EPMG. Therefore, discussion regarding the clinical implications of these interventions has been limited.

Currently, surgical reduction of retroglossal airway obstruction is a common method for treating tongue base collapse. Effect parameters (AHI, lowest O_2_ saturation, and ESS score) were used in the present study to assess pre- and post-operation conditions. The information provided by each article was not sufficient to perform a meta-analysis of surgical characteristics. Only three articles provided operation time data^11,12,16^. No significant differences were observed in operation times among TORS, CTBR, and radiofrequency (p > 0.05). However, when performed by an experienced surgeon with a low docking times, TORS was less time consuming than the other interventions. We also identified no significant differences in the success and complication rates of TORS and other surgical interventions for obstructive sleep apnea (p > 0.05). The volume of the tongue base is considered to be unrelated to surgical outcomes if Sher’s criteria are used (post-operative AHI < 20 and > 50% reduction of pre-operative AHI). However, the AHI and lowest O_2_ saturation displayed larger improvements after TORS than other interventions. The TORS and CTBR methods of tongue-base reduction exhibited competitive surgical results^26^. Furthermore, no significant differences in ESS score, lowest O_2_ saturation, operation time, or complication rate were observed between the two methods. A previous study revealed increased O_2_ saturation and a reduction in AHI after treatment with TORS, however, the study also reported that TORS resulted in a higher pain score^27^. Hoff et al. reported that the TORS procedure had a shorter operation times than other interventions^28^. Studies on CTBR reported comorbidities of bleeding, pain, transient dysphagia, and dysgeusia^29,30^. Studies reported a taste disturbance rate of 13.8–17.5% following CTBR, comparable to the reported dysgeusia rates in three systematic review studies on TORS^29,31,32^. We determined that TORS led to a higher AHI reduction even with a higher baseline in some studies^6,13,26^. However, the TORS still yielded results were comparable to those of other methods such as CTBR or CO_2_ laser surgery. TORS has been demonstrated to lower the AHI and reduce the severity of sleep apnea by reducing tongue base volume. However, the volume of tongue reduction is not proportional to the AHI reduction in each patient. A tailor-made surgical strategies and personalized treatments should be considered for each patient with sleep apnea. For patients with severe obstructive sleep apnea, surgical methods, such as TORS or CTBR, are a viable option for tongue base management. TORS is beneficial in terms of increasing O_2_ saturation to 90%, reducing the ESS score, not worsening comorbidities, and reducing comparative operation time. Furthermore, this surgery is preferable for drug-induced sleep endoscopy grade III–IV lingual tonsil hypertrophy. Vicini et al. reported that the resection tissue volume was significantly correlated with surgical success^33^. However, other studies have reported that the resection tissue volume is not related to surgical success but that creating a relatively stable channel for airway flow is crucial to reducing obstruction severity^27,34^. Therefore, although Vicini et al. suggested resection volume as a factor indicating surgical success^33^, smaller resection volumes can also lead to a significant reduction in the AHI and increase in the lowest O_2_ saturation.

Furthermore, more favorable prolonged and sustained airway patenting results could be achieved using the TORS approach compared with radiofrequency (higher recurrence rate because of tissue recovery response) and CTBR (tissue firewall response hinders the resection tongue base volume). High magnification and precision was also possible using TORS, improving the surgical procedure and hemostatic control^35^. Moreover, we believe that the cost-effectiveness of TORS is more favorable than that of other methods. Because of its once for all characteristic and one time multilevel though out surgery, reducing the need for surgical salvage and postoperative continuous positive airway pressure salvage, which increases the effectiveness and reduces the cost of therapy. Friedman et al. indicated that TORS is more expensive but also more efficacious for obstructive sleep apnea than other surgeries^6^. Furthermore, the use of robotic surgery offers improved surgical views during surgery, involves less working trauma to the patient, and adversely affects the health of surgeons less. For instance, the risk of a cervical and lumbar spine condition or wrist injury is lower compared with other traditional intraoral surgical methods. Therefore, TORS is a valuable alternative method for sleep surgeons and patients with obstructive sleep apnea and tongue base problems. The information obtained from the included articles provided numerous insights. However, the present study had limitations. First, most of the articles included were retrospective case studies that lacked details regarding experimental research design, resulting in a high risk of bias in the studies used in our systematic review. Second, TORS is an innovative example of technology-assisted medical treatment. However, the operation time was the only surgical parameter observed in the meta-analysis. Other surgical characteristic parameters were not sufficiently defined in the selected articles, which further limited comparison between TORS and alternative interventions.

## Conclusion

Our systematic review revealed that the use of TORS to treat obstructive sleep apnea had the same success and failure rates as other compared interventions with no statistical difference in operation times. The clinical effects on the AHI, lowest O_2_ saturation, and ESS score of treatment with TORS were comparable to the effects of alternative surgeries. In addition to TORS, numerous other methods are available for treating obstructive sleep apnea, such as CTBR and radiofrequency.

## Data Availability

Data supporting the findings of the current study are available from the corresponding author on reasonable request.

## References

[1] Maspero C, Giannini L, Galbiati G, Rosso G, Farronato G (2015). Obstructive sleep apnea syndrome: A literature review. Miner. Stomatol..

[2] Hou T, Hu S, Jiang X (2012). Tongue coblation via the ventral approach for obstructive sleep apnea-hypopnea syndrome surgery. Laryngoscope.

[3] de Bonnecaze G, Vairel B, Dupret-Bories A, Serrano E, Vergez S (2018). Transoral robotic surgery of the tongue base for obstructive sleep apnea: Preliminary results. Eur. Ann. Otorhinolaryngol. Head Neck Dis..

[4] Karaman M (2017). Comparison of fiber delivered CO_2_ laser and electrocautery in transoral robot assisted tongue base surgery. Eur. Arch. Otorhinolaryngol..

[5] Cammaroto G (2017). Tongue reduction for OSAHS: TORSs vs coblations, technologies vs techniques, apples vs oranges. Eur. Arch. Otorhinolaryngol..

[6] Friedman M (2012). Transoral robotic glossectomy for the treatment of obstructive sleep apnea-hypopnea syndrome. Otolaryngol. Head Neck Surg..

[7] Sutherland K, Chan ASL, Ngiam J, Darendeliler MA, Cistulli PA (2018). Qualitative assessment of awake nasopharyngoscopy for prediction of oral appliance treatment response in obstructive sleep apnoea. Sleep Breath.

[8] Albdah AA (2019). The impact of drug-induced sleep endoscopy on therapeutic decisions in obstructive sleep apnea: A systematic review and meta-analysis. Cureus.

[9] Semelka M, Wilson J, Floyd R (2016). Diagnosis and treatment of obstructive sleep apnea in adults. Am. Fam Physician.

[10] Tanna N (2016). Surgical management of obstructive sleep apnea. Plast. Reconstr. Surg..

[11] Hwang CS (2018). Comparison of robotic and coblation tongue base resection for obstructive sleep apnoea. Clin. Otolaryngol..

[12] Babademez MA, Gul F, Sancak M, Kale H (2019). Prospective randomized comparison of tongue base resection techniques: Robotic vs coblation. Clin. Otolaryngol..

[13] Lan WC, Chang WD, Tsai MH, Tsou YA (2019). Trans-oral robotic surgery versus coblation tongue base reduction for obstructive sleep apnea syndrome. PeerJ.

[14] Huntley C (2019). Comparing upper airway stimulation to transoral robotic base of tongue resection for treatment of obstructive sleep apnea. Laryngoscope.

[15] Yu JL, Mahmoud A, Thaler ER (2019). Transoral robotic surgery versus upper airway stimulation in select obstructive sleep apnea patients. Laryngoscope.

[16] Aynacı E, Karaman M, Kerşin B, Fındık MO (2018). Comparison of radiofrequency and transoral robotic surgery in obstructive sleep apnea syndrome treatment. Acta Otolaryngol..

[17] Folk, D., & D'Agostino, M. Transoral robotic surgery vs. endoscopic partial midline glossectomy for obstructive sleep apnea. World J Otorhinolaryngol Head Neck Surg 3,101–105(2017). 10.1016/j.wjorl.2017.05.004 PMC5683621 29204587

[18] Heiser C, Edenharter G, Bas M, Wirth M, Hofauer B (2017). Palatoglossus coupling in selective upper airway stimulation. Laryngoscope.

[19] Vanderveken OM (2013). Evaluation of drug-induced sleep endoscopy as a patient selection tool for implanted upper airway stimulation for obstructive sleep apnea. J Clin Sleep Med.

[20] Babademez MA (2010). Low-temperature bipolar radiofrequency ablation (coblation) of the tongue base for supine-position-associated obstructive sleep apnea. ORL. J .Otorhinolaryngol. Relat. Spec..

[21] Guilleminault C, Chervin R, Palombini L, Powell N (2000). Radiofrequency(pacing and thermic effects) in the treatment of sleep-disordered breathing. Sleep.

[22] Powell NB (1998). Radiofrequency volumetric tissue reduction of the palate in subjects with sleep-disordered breathing. Chest.

[23] Choussein S, Srouji SS, Farland LV, Gargiulo AR (2015). Flexible carbon dioxide laser fiber versus ultrasonic scalpel in robot-assisted laparoscopic myome. J. Minim. Invas. Gynecol..

[24] Remacle M (2012). Combining a new CO_2_ laser wave guide with transoral robotic surgery: A feasibility study on four patients with malignant tumors. Eur. Arch. Otorhinolaryngol..

[25] Murphey AW, Kandl JA, Nguyen SA, Weber AC, Gillespie MB (2015). The effect of glossectomy for obstructive sleep apnea: A systematic review and meta-analysis. Otolaryngol. Head Neck Surg..

[26] Lee JM, Weinstein GS, O'Malley BW, Thaler ER (2012). Transoral robot-assisted lingual tonsillectomy and uvulopalatopharyngoplasty for obstructive sleep apnea. Ann. Otol. Rhinol. Laryngol..

[27] Cammaroto G (2018). Trans-oral robotic tongue reduction for OSA: Does lingual anatomy influence the surgical outcome?. J. Clin. Sleep Med..

[28] Hoff PT, Glazer TA, Spector ME (2014). Body mass index predicts success in patients undergoing transoral robotic surgery for obstructive sleep apnea. ORL J. Otorhinolaryngol. Relat. Spec..

[29] Justin GA, Chang ET, Camacho M, Brietzke SE (2016). Transoral robotic surgery for obstructive sleep apnea: A systematic review and meta-analysis. Otolaryngol. Head Neck Surg..

[30] Lin HC, Hwang MS, Liao CC, Friedman M (2016). Taste disturbance following tongue base resection for OSA. Laryngoscope.

[31] Miller SC, Nguyen SA, Ong AA, Gillespie MB (2017). Transoral robotic base of tongue reduction for obstructive sleep apnea: A systematic review and meta-analysis. Laryngoscope.

[32] Meccariello G (2017). Transoral robotic surgery for the management of obstructive sleep apnea: a systematic review and meta-analysis. Eur. Arch. Otorhinolaryngol..

[33] Vicini C (2010). Transoral robotic tongue base resection in obstructive sleep apnoea-hypopnoea syndrome: A preliminary report. ORL J. Otorhinolaryngol. Relat..

[34] Bahgat A (2020). Transoral endoscopic coblation tongue base surgery in obstructive sleep apnea: Resection versus ablation. ORL J. Otorhinolaryngol. Relat. Spec.

[35] O'Malley BW, Weinstein GS, Snyder W, Hockstein NG (2006). Transoral robotic surgery (TORS) for base of tongue neoplasms. Laryngoscope.

